#  Effect of Charge on Separation of Liposomes upon Stagnation 

**Published:** 2017

**Authors:** Mahsa Narenji, Mohammad Reza Talaee, Hamid Reza Moghimi

**Affiliations:** a *Department of Pharmaceutics and Nanotechnology, School of Pharmacy, Shahid Beheshti University of Medical Sciences, Tehran, Iran. *; b *Department of Railway Engineering Rolling Stock, Iran University of Science and Technology, Tehran, Iran.*

**Keywords:** Liposomes, Charge, Zeta potential, Particle size, Sedimentation, Storage, Separation

## Abstract

Liposomes are used widely as drug delivery systems in different forms including nanosuspensions, osmotic pumps, infusion pumps, and IV injections. Some of these systems (e.g. infusion or osmotic pumps) might stay stagnant for a long time during or before administration, and therefore, might face phase separation. In spite of these, there are no data available about the behavior of liposomal systems upon stagnation in such drug delivery systems. As a part of a series of investigations on convective flow and stagnation of liposomes, the current work represents the effects of charge on liposomes separation upon stagnation.

Positive, negative, and neutral liposomes, with zeta potentials of +56, -50 and 1.4 mV respectively, were prepared and encountered gravity (separating force) in a designed sedimentation model. Samples were collected over 25 h and their D0.5 (diameter which half of the particles are smaller than), particle size distribution, and phospholipid contents were evaluated. The ratio of the D0.5 in the last to the first sample, (Separation Factor) for positive, negative, and neutral liposomes were calculated to be 1.00 (no separation), 0.98 (no separation), and 0.33 (separation) respectively. The same trend was observed for lipid contents and particles population. These data show that liposomes’ charge affect their separation under gravity and is a very important factor in their uniformity upon storage, pre-administrational steps, and even during administration in systems such as infusion pumps.

## Introduction

Among the several drug delivery systems, liposomes have drawn a lot of interest as advanced and versatile carriers for pharmaceuticals. At present, liposomal formulations span multiple areas, from clinical application of the liposomal drugs to the development of various multifunctional liposomal systems for therapy and diagnostics (1). The special properties of liposomes have generated numerous applications of liposomes as drug delivery applications (2) which are feasible with various drug molecules and routes of administration and as models for biological membranes (3). The application of liposomes are growing rapidly as complex and sophisticated liposomal formulations such as dendrosomes (4, 5) and magnetoliposomes (6, 7) are being introduced in the field. The stability of liposomes is a major consideration in all steps of their production and administration: from process steps to storage to delivery. When a pharmaceutical dosage form is altered the stability of the drug may be changed. A stable dosage form maintains its physical integrity and does not adversely influence the chemical integrity of the active ingredient during its life on the shelf (8). Different instabilities such as chemical and physical are probable to occur. One of these instabilities is changes in particle size distribution of liposomal formulations. Exact knowledge of the sizes of these nanoparticles is essential because, size can substantially affect physicochemical and biopharmaceutical behavior of the formulation. For example, variations in particle size can affect drug release kinetics, transport across biological barriers, and pharmacokinetics in the human body (9). This study focuses on the influence of physical factors in size separation of liposomes –a physical instability- as a respond to gravitational force during stagnation. 

Particles size distribution of liposomes can change in stagnating conditions such as: in storage and mixing tanks, intravenous infusion pumps or drip, osmotic or physically actuated pumps, etc. Size separation of particles encountering gravity can be affected by their physical characteristics and the media they are dispersed in. Some studies have reported the possible effect of charge on the liposomal formulations’ stability or deposition. Murugova and Balavy, reported that presence of charged DOPS in liposomal formulations prevents the vesicles from sedimentation (10). Chow *et al.,* 2007 also named charge as an effective factor in lung deposition of particles (11). Other studies have suggested that coalescence of liposomes increases in neutral charges, due to the natural tendency of aggregation in non-charged liposomes (12, 13). None of the above studies have investigated the influence of charge on liposomes sedimentation as the only effective factor mechanistically. Although these are well studied for large particles > 0.5µ and claimed to be less important for nano-sized particles (14), but actually not well investigated for the latter group. Such problems can worsen by introduction of novel complex liposomal such as dendrosomes (4, 5) and magnetoliposomes (6, 7) that are expected to be heavier due to their heavy loads. As a part of series of studies aimed to investigate the behavior of liposomes in convective flow and stagnation, this article investigates the influence of the liposomes’ charge on their separation during stagnation. Such stagnations are possible in liposomal drug delivery systems both before administration (e.g. during storage and pre-administration steps) and during administration (e.g. in a running infusion pump). There is not such a data available in the literature.

## Experimental

*Material and Methods*

*Materials*

1,2-distearoyl-sn-glycero-3-phosphocholine (DSPC, > 99%), 1,2-distearoyl-sn-glycero-3-phospho-(1ʹ-rac-glycerol) (DSPG, >99%) and 1,2-dioleoyl-3-trimethylammonium-propane (DOTAP, > 99%) were purchased from Lipoid GmbH (Germany), cholesterol (>99%) was purchased from Sigma (USA), methanol (99%), chloroform (99.4%), ammonium thiocyanate (98.5%) and ferric chloride hexahydrate (> 99%) were purchased from Merck (USA). All other chemicals were of pharmaceutical grade.

*Preparation liposomal formulations*

The liposomal formulation was prepared using lipid film hydration method. In brief, the lipid phase with total concentration of 20 mM was dissolved in chloroform: methanol mixture (2:1). The organic solvent was evaporated at 60 ⁰C under the vacuum using a rotary evaporator (Heidolph, Germany), to form a lipid film. The film was then hydrated by deionized water at the same temperature. Extrusion (Northern Lipids, Canada) of liposomes through 1µM polycarbonate filter (Sigma, USA) was conducted to reduce their size. 

Different lipid compositions were used to obtain neutral, positive, and negatively charged liposomal formulations. Neutral liposomes were formulated using DSPC: cholesterol (70:30 molar ratios). The lipid phase of negatively charged liposomes was consisted of DSPC: DSPG: cholesterol (65:10:25 molar ratios) and the positively charged liposomes were prepared using DSPC: DOTAP: cholesterol (65:10:25 molar ratios). All the other processes in all the three formulations were the same. As is seen in the formulations, the compositions of these three systems are very close except for the charged lipids. This similarity together with similar particle size distribution of liposomal formulations prevents contribution of interfering factors, other than the charges, in the separation of liposomes.

*Characterization of nanoparticles*

*Measureing the size distribution and zeta potential*

Size distributions of the particles were measured using Mastersizer 2000 (Malvern, UK). Charges of the nanoparticles were confirmed by measuring the zeta potential of the liposomal formulations, using Nanozetasizer (Malvern, UK). D0.5 (diameter which size of the 50% of the particles is less than) was also measured and used later.

*Measurement of phospholipid content of liposomes*

As an indicator of liposomes concentration, the concentration of phospholipids was measured using the Stewart Method (15). This method uses the reaction between phosphate group of phospholipids and ammonium ferrothiocyanate to measure the concentration of phospholipid in the samples. In brief, a proper amount of liposome was dissolved in chloroform and was shaken vigorously in the presence of ammonium ferrothiocyanate. The concentration of phospholipid the chloroform was then measured using UV-Visible spectrophotometer (CECIL, Italy) at 488 nm after separation of chloroform from aqueous phase.

*Separation studies*

Possible separation of liposomes upon stagnation was investigated in burettes in a vertical position using the gravity as the driving force. To perform this investigation, liposomal formulations were first put in separate burettes and after a settling time of 15 h, samples were collected in defined time interval for 10 h. Such a time protocol, that was chosen based on our preliminary studies, was found to be suitable for observation of separation and near completion of this process. Changes in the formulations during the experiment were then analyzed using size distribution profile, D0.5, zeta potential and lipid contents of samples. Please note that this investigation is not a stability test and therefore longer times or chemical stability analyses were not performed here.

*Statistical analysis*

Statistical analysis of the results was performed by one-way ANOVA test using the SPSS Statistics 17.0 software (SPSS Inc., USA).

## Results and Discussion

*Characterization of liposomes*

**Figure 1:**
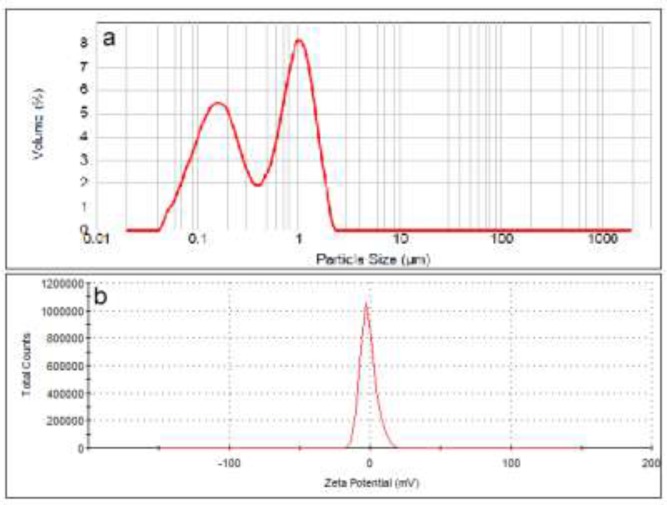
Particle size distribution (a) and zeta potential (b) of neutral liposomes (DSPC:cholesterol).

The particle size distribution curve of neutral liposomes showed two peaks at 200 and 1100 nm with particle range between 30-2000 nm. Zeta potential of this formulation was measured to be -1.38 mV (Figure 1) and its D0.5 was measured to be about 450 nm with a span of 3.1 (n = 3).

**Figure 2:**
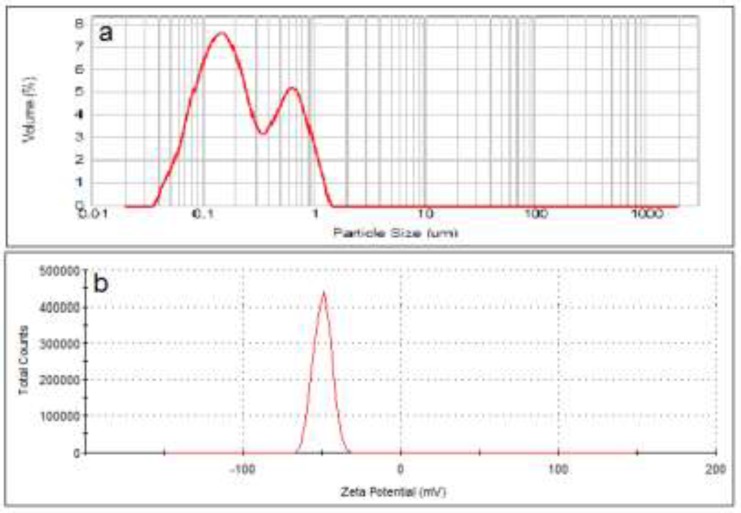
Particle size distribution (a) and zeta potential (b) of anionic liposomes (DSPC: DSPG: cholesterol

The negatively charged liposomes showed a D0.5 of about 200 nm with a span of 3.5 (n = 3). Range of size distribution in this formulation was from 30 nm to 1600 nm. The size distribution curve contained two peaks at 180 and 650 nm. Analyzing the zeta potential of these particles confirmed that they carry a negative charge of about -50 mV (Figure 2).

**Figure 3:**
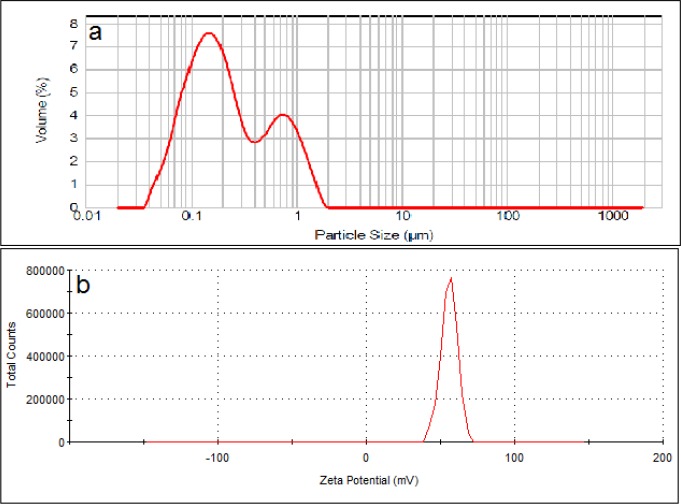
Particle size distribution (a) and zeta potential (b) of cationic liposomes (DSPC: DOTAP: cholesterol

Particle size of the positively charged liposomes showed D0.5 of about 200 nm with a span of 4.2 (n = 3). Size distribution curve of these particles contains two peaks at 180 and 750 nm that covers range of between 30-1600 nm. Zeta potential of the particles measured to be about +56 mV (Figure 3).

To investigate the effect of charges on liposomes separation, the particle size of the liposomal formulations was adjusted to be as similar as possible (Figure 1-3) while their zeta was different from each other. Also the lipid composition of the positively charged, negatively charged and neutral liposomes was almost the same, except for the charge determining lipids. Particle size distribution of all liposomal formulations were intentionally chosen to be wide, hence high spans, to have heterogeneous samples and higher possibility of size separation upon stagnation. The chosen size ranges cover the sizes usually employed or investigated in pharmaceutical studies and also covers the particle with high and low chances of Brownian motion.

**Figure 4:**
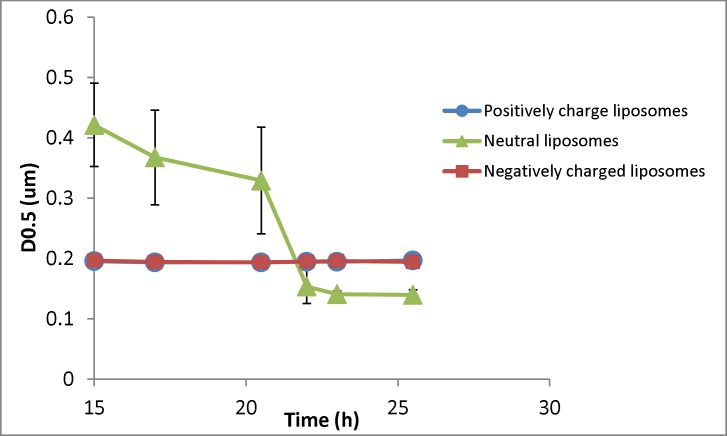
Changes in the particle size (D0.5) of liposomal formulation with different charges (n = 3) for samples taken at different times (resembling different depth in the burette). The zeta potential of initial (freshly prepared liposomes) are -50, -1.4 and +56 mV for negative, neutral and positive formulation respectively and their particle size range is 30-1600nm (negative liposomes), 30-2000 (neutral liposomes) and 30-1600 (positive liposomes

**Figure 5:**
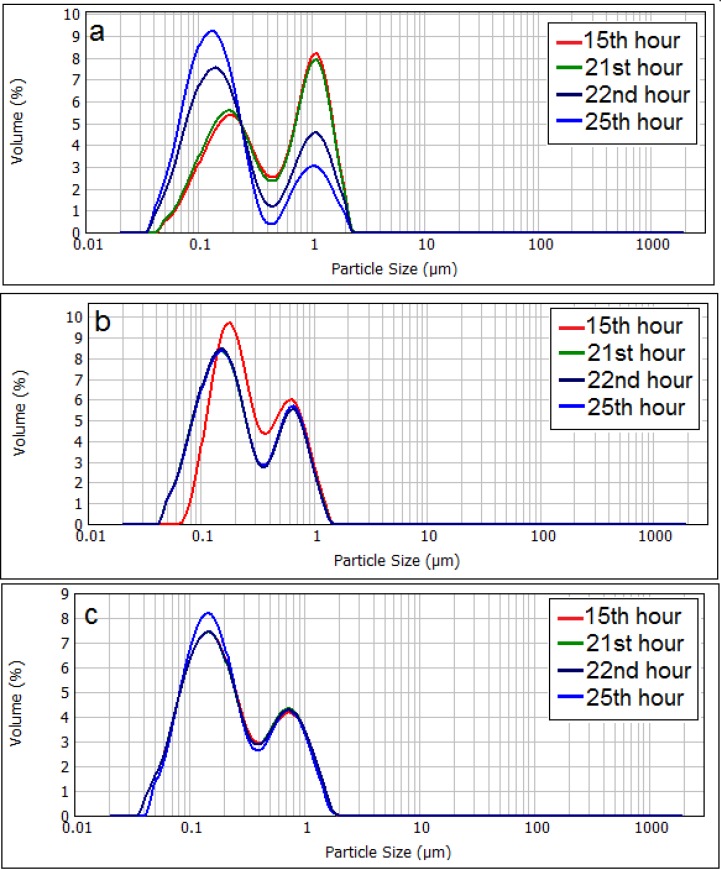
Changes in the particle size distribution of neutral liposomes (a), negatively charged (b), and positively charged (c) liposomes. The zeta potential of initial (freshly prepared liposomes) are -50, -1.4 and +56 mV for negative, neutral and positive formulation respectively and their particle size range is 30-1600nm (negative liposomes), 30-2000 (neutral liposomes) and 30-1600 (positive liposomes

**Figure 6:**
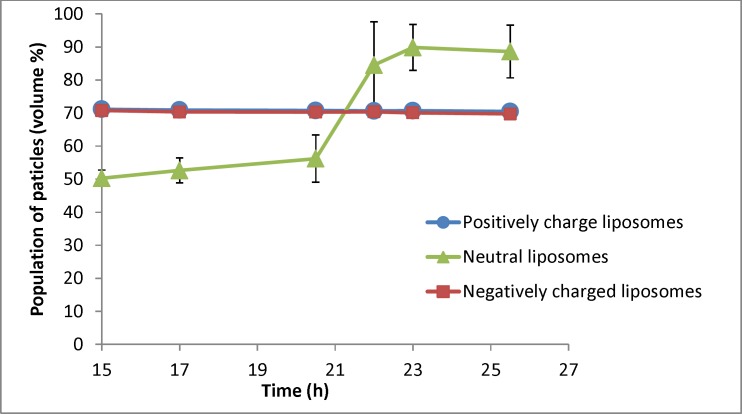
Changes in population of particles smaller than 400nm for liposomal formulation with different charges (n = 3) for samples taken at different times (resembling different depth in the burette). The zeta potential of initial (freshly prepared liposomes) are -50, -1.4 and +56 mV for negative, neutral and positive formulation respectively and their particle size range is 30-1600nm (negative liposomes), 30-2000 (neutral liposomes) and 30-1600 (positive liposomes

**Figure 7:**
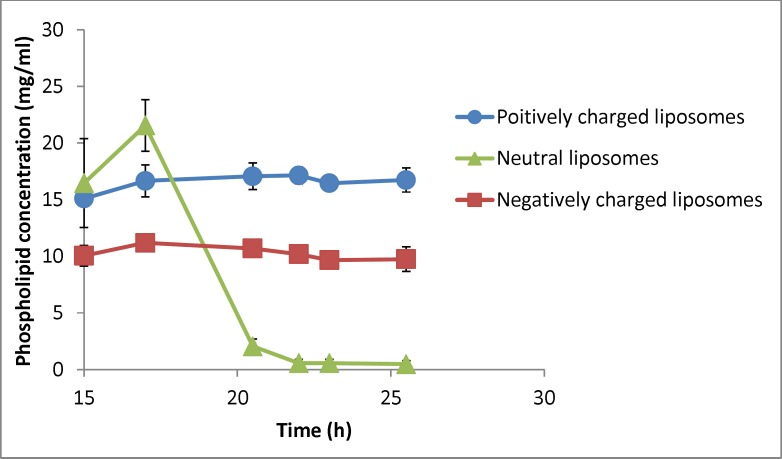
Changes in phospholipid concentration in liposomal formulation with different charges under gravity over time (n = 3). The zeta potential of initial (freshly prepared liposomes) are -50, -1.4 and +56 mV for negative, neutral and positive formulation respectively and their particle size range is 30-1600nm (negative liposomes), 30-2000 (neutral liposomes) and 30-1600 (positive liposomes

*Separation of liposomes with different charges*

Figure 4 shows the changes of D0.5 of three liposomal formulations encountering the gravity. The negatively and positively charged formulations showed no changes of particle size over time and among different samples, while the particle size of neutral liposomes got smaller over time. The size of neutral liposomes changed from 420 nm (15th h) to 140 nm (26^th^ hour). The separation factor (D0.5_last sample_/D0.5_first sample_) for this formulation was measured to be 0.33, while the same factor was measured to be 1.005 and 0.989 for positively and negatively liposomes respectively showing no size separation over time for charged liposomes. Please note that the sampling time resembles the sample depth as well.

The Stoke’s law discusses the free falling of particles at a constant rate and without any hindrance and uses the Anderson apparatus to measure the diameter of particles (14) which is close to the sedimentation method of the current study. However, our model has employed narrower system and therefor can work with lower volume at the same depths. Our model provides much higher height as small volumes.

The main necessity for this law for the particles is absence of aggregation and inter-particular interactions such as electrostatic interactions. These interactions are more noticeable in particle with smaller size which is also the subject of present investigation. Sedimentation of nanoparticles smaller than 500 nm is almost negligible due to Brownian motion. Results of the present study show that sedimentation of particles occur even at sizes lower than what is supposed to be Brownian motion border (Figure 4), probably due to aggregation or flocking. Our results also indicate that even large particles do not sediment in charged liposomes.

Particle size distribution curve of neutral liposomes shows that population of smaller sized liposomes grows significantly over time (Figure 5), whilst the size distribution of charged liposomes showed no or little change during the experiment and the size distribution of charged liposomes remained almost unchanged (P > 0.05).

Figure 6 shows the percentage of particles smaller than 400 nm for different formulations. As shown in Figure 6, there is no change for charged liposomes, while in neutral liposomes the population of liposomes less than 400 nm increases over time. The separation factor for this parameter in neutral, negative and positive liposomal formulation has been calculated as 1.76, 0.98 and 0.99 respectively. These results indicate that percentage of neutral liposomes with sizes smaller than 400 nm at the 26th hour is almost 2 times greater than the 15th h.

The above results showed that sedimentation of neutral liposomal formulations, even below the Brownian motion’s border, is considerable and there is a growth in nanoparticles population over time and in higher depths. Charged liposome acted completely different from the neutral particles and no sedimentation and size reduction occurred over time although they contain particles as large as 2 µM. This could be the effect of electrostatic repulsion between the particles which inhibited the sedimentation and separation of particles, and therefor size distribution of charged particles remained the same in all depths.

Gravitational, van der Waals and buoyancy are known to be three main forces in a suspension of particles. Van der Waals is the main force to influence the stability of these dispersions. If two particles approach each other up to a minimum distance, this force will bind them together and can cause sedimentation and instability of dispersion (16). Increasing the charge density of these dispersions, which is known as electrostatic stabilization, prevents this phenomenon (14). Regarding the present results, it seems that the electrostatic repulsion between the charged liposomal particles conquers gravity and prevents them from sedimentation, so the charged formulations remain uniformly distributed during the experiment. Lack or shortage of the electrostatic forces in the neutral formulation makes gravity the dominant forces in the system. In such conditions, any size separation (e. g. sedimentation) in the formulation led to increased percentage of smaller particles in the collected samples by increasing the time of sampling.

There is not such a data available on the effects of charge on separation of liposomes upon stagnation. However, Plessis *et al.* (17) investigated long-term (6 months) physical stability of different liposomes with different lipid compositions with positive zeta potentials of 75-100 mV. Their results showed that presence of cationic lipids decreases long-term physical stability of liposomes. They did not study phase separations upon stagnation or effect of charge on this phenomenon. The reported increased instability due to positive lipids might be related to higher chemical interaction of charged molecules. Other studies have suggested charge to be a promising factor on stability of liposomes (12, 13) but there is lack of data about the effect of charge on their stagnation and separation behavior in such conditions.

Changes in the phospholipid concentration during the experiment were also investigated as an indicator of liposomes’ concentration (Figure 7). Statistical analysis showed that phospholipid concentration of neutral liposomes in each time point was significantly different from the other samples (P < 0.05) and there was no difference in phospholipid concentration of charged liposomes during the experiment (P > 0.05). Separation factor for this parameter in neutral, negative and positive liposomal formulations encountering gravity was measured to be 0.03, 0.96 and 1.11. These results indicate that the charged particles with different sizes do not tend to separate under gravity force, whereas the neutral particles tend to behave the opposite way and the system cannot be considered homogenous considering the lipid composition.

These results show the importance of liposome formulation properties and indicate that the charge of liposomes is an effective factor on their sedimentation over time. The above result could be used in development new liposomal formulations, storage protocols, application considerations such as in infusion set or pumps, osmotic pumps, etc. Such separations are expected to affect other properties such as rheology of the system and also expected to be much more evident for heavy formulations such as magnetoliposomes (7).

The findings of the present investigation can be used together with other of liposomal stabilization methods such as liposomal gel formulation (18, 19). The results of the present investigation also indicate that instability can occur in short times and can be very important in application of liposomes such as in infusion systems.

## conclusion

This study shows that nanoparticles with different surface charges do not behave the same when encounter gravity as a separating force. Neutral liposomes showed separating behavior under gravity, up to three times change in the particle size over a short time, whereas the charged particles with the same properties did show separation. This indicates that charge is a very important factor on the stability of these particles upon stagnation. Presence of positive or negative charges on the surface of nanoparticles restricts sedimentation of liposomes, even for particles sizes below the supposed border for the Brownian motion. Results of the present investigation also indicate that the instability can occur in short times and can be very important in application of liposomes such as in infusion systems or osmotic pumps. The findings of the present investigation can be used in formulation of stable liposomes, alone or together with other methods of stabilization. Further studies are in progress in our laboratories to investigate the effect of convective flows on liposomal separations.
